# Computational Modeling and Experimental Approaches for Understanding the Mechanisms of [FeFe]‐Hydrogenase

**DOI:** 10.1002/advs.202408297

**Published:** 2025-05-08

**Authors:** Chang‐Ah Kim, Jiabin Wu, Jun Zhu, Huaiguang Li, Zhihai Ke

**Affiliations:** ^1^ School of Science and Engineering Shenzhen Key Laboratory of Innovative Drug Synthesis The Chinese University of Hong Kong Shenzhen Guangdong 518172 P. R. China

**Keywords:** catalyst development, density functional theory calculations, [FeFe] hydrogenases, hydrogenases, nature‐inspired design

## Abstract

Learning from nature has emerged as a promising strategy for catalyst development, wherein the remarkable performance of catalysts selected by nature over billions of years of evolution serves as a basis for the creative design of high‐performance catalysts. Hydrogenases, with their exceptional catalytic activity in hydrogen oxidation and production, have been employed as prototypes for human learning to achieve better catalyst design. A comprehensive understanding of hydrogenases' structures and catalytic mechanisms is crucial to replicate and exceed their performance. Computational modeling has proven to be a powerful tool for elucidating the reduction chemistry of [FeFe]‐hydrogenases. This review overviews recent computational and experimental efforts, focusing on density functional theory (DFT) calculations applied to [FeFe] hydrogenases. It summarizes current knowledge on identifying active sites in [FeFe] hydrogenases and the reaction cycles involved in hydrogen metabolism.

## Graphical abstract

A comprehensive understanding of hydrogenases' structures and catalytic mechanisms is crucial to replicate and exceed their performance. Computational modeling has proven to be a powerful tool for elucidating the reduction chemistry of [FeFe]‐hydrogenases. This review overviews recent computational and experimental efforts, focusing on DFT calculations applied to [FeFe] hydrogenases. It summarizes current knowledge on identifying active sites in [FeFe] hydrogenases and the reaction cycles involved in hydrogen metabolism.

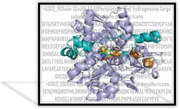

## 1 Introduction

Bio‐inspired catalysis has emerged as a promising avenue to drive efficient and clean energy conversion processes in the quest for sustainable energy sources.^[^ ^1^, ^2^, ^3^ ^]^ Among the biocatalysts found in nature, hydrogenases, specifically [FeFe]‐hydrogenases, have been found to exhibit remarkably high turnover activities for both hydrogen (H_2_) release and H_2_ oxidation, notably at neutral pH, ambient temperatures, and negligible electric overpotential.^[^ ^4^, ^5^ ^]^ Due to these merits, [FeFe]‐hydrogenase represents the “gold standard” in enzymatic hydrogen turnover.^[^ ^4^, ^6^, ^7^, ^8^ ^]^ Its exceptional ability to catalyze the reversible conversion of molecular hydrogen has inspired the development of novel renewable energy production and storage approaches. In addition, these properties make [FeFe] hydrogenases a promising candidate for applications such as electrode interfacing in hydrogen fuel cells, hydrogen production,^[^ ^9^ ^]^ and potential combinations with cascade reactions for CO_2_ conversion or cofactor regeneration (like NADPH), facilitating product separation in various biotechnological processes.^[^ ^6^ ^]^

**Figure 1:**
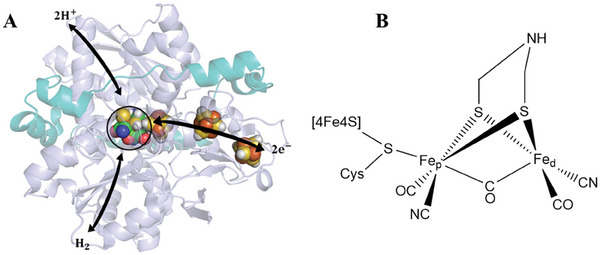
Schematic representation of crystal structure A) and active site B) of [FeFe] hydrogenase from *Desulfovibrio Desulfuricans* (PDB ID: 6SG2). This enzyme consists of two subunits (Small and large chains are colored cyan and purple, respectively). The large subunit of [FeFe]‐hydrogenases contains the chain of electron transfer, hydrogen evolution, and hydrogen transfer processes. Additionally, the catalytic active site is denoted by a circular marking. Reproduced with permission.^[^ ^12^ ^]^ Copyright 1999, Cell Press.

Since Stephenson and Stickland reported enzymatic hydrogen activation in 1931,^[^ ^3^ ^]^ [FeFe]‐hydrogenases, derived from diverse microorganisms, have served as model enzymes for studying the catalytic mechanisms of hydrogen conversion.^[^ ^10^ ^]^ They feature an intricate active site, the hydrogen‐activating “H‐cluster,” comprised of an iron‐iron ([FeFe]) binuclear center coordinated by a unique array of iron and sulfur atoms, the bridged ligand, π‐accepting ligands cyanide (CN^−^) and carbon monoxide (CO) (**Figure** **1**).^[^ ^4^, ^11^, ^12^, ^13^ ^]^ The structure and catalytic mechanism of [FeFe] hydrogenase have been extensively studied, and great progress has been achieved in the past few decades.^[^ ^14^, ^15^, ^16^, ^17^, ^18^ ^]^ Still, some issues remain controversial and, in some cases, are serious and have not been resolved to date.^[^ ^8^, ^19^, ^20^ ^]^ Computational techniques have emerged as powerful tools in exploring and understanding complex biological systems, enabling researchers to unravel the intricate details of enzymatic mechanisms. The computational study of [FeFe] hydrogenase has become indispensable in deciphering the underlying principles governing its exceptional catalytic activity.^[^ ^21^, ^22^, ^23^ ^]^ By employing computational approaches, researchers can investigate complex reaction pathways, probe the electronic structures of intermediates, predict the thermodynamics and kinetics of the catalytic cycle, and explore the impact of various factors on enzyme performance.^[^ ^21^, ^24^, ^25^ ^]^

This review aims to highlight the significance of computational‐experimental studies of [FeFe] hydrogenases, shedding light on the current state‐of‐the‐art methodologies and showcasing recent advances in H‐cluster and catalytic cycles. By bridging the gap between theory and experiment, computational studies of [FeFe] hydrogenase explore an exciting frontier in bio‐inspired catalysis and offer tremendous potential for the development of efficient and eco‐friendly energy conversion technologies.

## 2 Advances in Understanding the H‐Cluster

The application of X‐ray diffraction (XRD) to determine the crystal structure of [FeFe]‐hydrogenase from *C. pasteurianum* and *D. desulfuricans* has played a crucial role in establishing a detailed molecular understanding of hydrogen catalysis.^[^ ^26^, ^27^, ^28^, ^29^ ^]^ However, detailed insight into the dynamic H_2_ turnover at the H‐cluster depends on spectroscopy and quantum mechanical calculations.^[^ ^30^, ^31^, ^32^, ^33^, ^34^ ^]^ These studies include electron paramagnetic resonance (EPR) spectroscopic techniques, infrared spectroscopy, Mössbauer spectroscopy, nuclear resonance vibrational spectroscopy (NRVS), nuclear magnetic resonance spectroscopy (NMR) and computational modeling, etc.^[^ ^35^, ^36^, ^37^, ^38^ ^]^ It is revealed that the H‐cluster consists of a [4Fe–4S] cluster linked to a [2Fe] diiron site via a cysteine (Figure 1B).^[^ ^8^, ^11^, ^39^, ^40^ ^]^ The geometric and electronic properties of the [2Fe] subcluster are closely connected to the [4Fe–4S] subcluster by strong spin coupling exchange through this bridging cysteine ligand. An azadithiolate (ADT) ligand and a CO ligand bridge the two irons of the [2Fe] site.^[^ ^36^, ^39^, ^41^ ^]^ For the ADT ligand, energy in calculations indicates that the bridgehead N atom provides a suitable nitrogen base and a kinetically and thermodynamically favorable route for heterolytic cleavage or formation of dihydrogen.^[^ ^42^ ^]^ Later, experimental evidence, including ^14^N hyperfine interactions studied by Silakov et al.,^[^ ^42^ ^]^ revealed the chemical nature of the dithiolate group, whose central atom is a nitrogen atom.^[^ ^43^, ^44^ ^]^ The nitrogen base in the ADT ligand combines with the low valent Lewis acidic Fe site (Fe_d_) to create a unique configuration of a frustrated Lewis pair.^[^ ^45^ ^]^ This arrangement is ideal for efficient hydrogen splitting through heterolysis.^[^ ^19^, ^46^ ^]^

**Figure 2:**
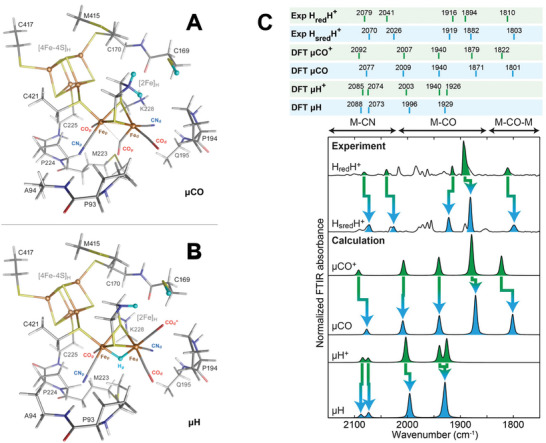
Optimized structures based on DFT calculations of µCO A) and µH B) models shown in tube representation. The ball representation indicates the Fe sites (brown) and the three H‐to‐D exchangeable protons (light blue). Single‐letter amino acid labels correspond to the *Cr*HydA1 enzyme sequence. Element colors are C (grey), H (white), N (blue), O (red), Fe (brown), and S (yellow). C) Experimental and DFT‐calculated IR spectra of the H_red_H^+^ and H_sred_H^+^ states in H_2_O. The calculated µCO and µCO^+^ spectra were generated from DFT calculations on a bridging CO model in (A). The µH and µH^+^ spectra were generated from DFT calculations on a bridging hydride model in (B). The peaks corresponding to the H_red_H^+^ and H_sred_H^+^ states are colored green and blue, respectively. Reproduced with permission.^[^ ^36^ ^]^ Copyright 2020, American Chemical Society.

Further, both irons are coordinated by terminal CO and CN^−^ ligands. Fourier transform infrared (FTIR) spectroscopy can address the CO/CN^−^ ‐stretching frequencies as intrinsic marker bands. Quantum chemical calculations are utilized to unravel the diatomic ligands and yield refined H‐cluster geometries specifically. The H‐cluster exhibits a distinct pattern of five bands in its active oxidized state, Hox. These bands correspond to the stretching vibrations of the CO and CN^−^ ligands at Fe_p_(II) and Fe_d_(I) sites, respectively. A lower frequency band is also associated with the Fe−Fe bridging carbonyl vibration (µCO).^[^ ^47^ ^]^ Low‐temperature FTIR measurements have confirmed the bridging CO ligands for H_red_H^+^ and H_sred_H^+^ states which are one and two electron reduced states of Hox respectively. DFT calculations are consistent with a bridging CO structure with a protonated ADT ligand than a structure in which a hydride was bridged between the two Fe ions of the [2Fe] subcluster (**Figure** **2**).^[^ ^36^ ^]^ The CO and CN^−^ ligands promote reversible heterolytic H_2_ cleavage and stabilize low spin states of the [2Fe] atoms.^[^ ^48^, ^49^ ^]^ The CN^−^ ligands further fine‐tune the energy levels of the frontier orbitals of the [4Fe–4S] and [2Fe] subcluster moieties, facilitating fast electron transfer between the two subclusters during the catalytic process.^[^ ^50^ ^]^ However, DFT calculations in some studies also indicated bridging hydride is a more favorable structure.^[^ ^15^, ^51^ ^]^ It necessitates further insights in the future from advanced models.

**Figure 3:**
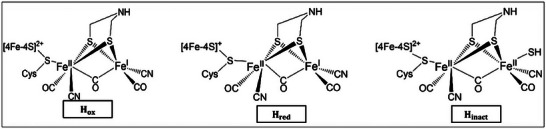
Proposed catalytic active site models in [FeFe] hydrogenase for H_2_ activation of the H‐cluster: H_ox_, H_red_, and H_inact_.

The infrared stretching frequencies of various geometry‐optimized structural candidates have been simulated through DFT calculations.^[^ ^52^, ^53^ ^]^ By comparing the experimentally determined and computed CO and CN stretching frequencies, they ruled out structural candidates with the Fe(III)Fe(III) and Fe(II)Fe(III) oxidation states.^[^ ^53^ ^]^ Furthermore, the model predicted that the best structural candidate for “overoxidized” H_inact_ state, H_ox_, and one electron reduced active H_red_ state corresponded to Fe(II)Fe(II), Fe(II)Fe(I), and Fe(II)Fe(I) complex, respectively (**Figure** **3**). For the H_ox_ state, there is an open site on the distal iron.^[^ ^53^ ^]^ The presence of Fe(I) in the catalytic cycle was considered a key to the efficiency of [FeFe]‐hydrogenase in forming hydrogen molecules. A subsequent computational study by Fiedler and Brunold confirmed the Fe^II^Fe^I^ nature of the H_ox_ and suggested including the proximal [4Fe–4S] cluster for the correct calculation of the EPR parameters.^[^ ^54^ ^]^ These states have been experimentally identified in the hydrogenase from *Desulfovibrio desulfuricans* (*Dd*) based on the EPR/ENDOR.^[^ ^55^ ^]^

## 3 Catalytic Cycle of [FeFe] Hydrogenases

Over the past two decades, theoretical and experimental methods have been extensively employed to gain deeper insights into the molecular details of H_2_ oxidation and H^+^ reduction at the di‐iron active site.^[^ ^56^, ^57^, ^58^, ^59^, ^60^, ^61^, ^62^, ^63^, ^64^ ^]^ Even so, the catalytic cycle of [FeFe] hydrogenases remains hotly discussed. Two main mechanisms of proton reduction in [FeFe]‐hydrogenase have been proposed based on computational modeling and experimental findings.^[^ ^8^, ^19^ ^]^

**Figure 4:**
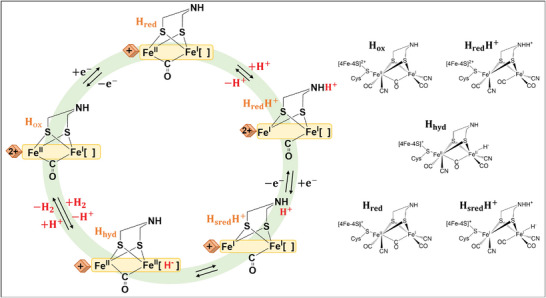
The catalytic cycle of [FeFe] hydrogenases. Here, H_ox_ is reduced to H_red_ by one electron at [4Fe‐4S], and proton‐coupled electronic rearrangement yields H_red_H^+^, which can then be further reduced to H_sred_H^+^. Isomerisation of H_sred_H^+^ yields H_hyd_, with a terminal hydride, which then reacts with a proton to form H_2_.

In the catalytic cycle of [FeFe] hydrogenases, the [4Fe‐4S] cluster experiences a cycle between the oxidized (2+) and reduced (1+) states, while the [2Fe] site undergoes transitions between a Fe(II)Fe(II) state, Fe(II)Fe(I) state, and Fe(I)Fe(I) state. The active oxidized state, H_ox_, is characterized by an oxidized [4Fe‐4S] site and a Fe(II)Fe(I) [2Fe] site (**Figure** **4**).

A mixture of two H_red_ states is formed upon reduction by one electron. In one state, [4Fe‐4S]H is reduced to the 1+ state, while in the other state, [2Fe] is reduced to the Fe(I)Fe(I) state. The former state, stable at high pH, is proposed to be a deprotonated form (H_red_), while the latter state, stable at low pH, is suggested to be a protonated form (H_red_H^+^) with the proton presumably located on the nitrogen of the ADT ligand. This is in line with the DFT predictions.^[^ ^42^, ^52^ ^]^ This protonation event stabilizes the Fe(I)Fe(I) configuration and allows further reduction of the [4Fe‐4S] site to the H_sred_H^+^ state. H_red_H^+^ and H_sred_H^+^ states contain sufficient electrons and protons to form a terminal hydride on Fe_d_, resulting in states referred to as H_hyd:ox_ and H_hyd:red_, respectively. Both states have an Fe(II)Fe(II)[2Fe] site, but H_hyd:red_ also possesses a reduced [4Fe‐4S] site. Investigations on synthetic molecular catalysts point to the intermediacy of terminal hydrides, which interact with the pendant amine of the ADT ligand.^[^ ^65^ ^]^ Recent studies have observed H_hyd_ states in both wild‐type and hybrid [FeFe]‐hydrogenases.^[^ ^35^, ^66^, ^67^ ^]^ DFT calculations have confirmed the spectral features of an iron‐bound terminal hydride and have shown that the vibrational frequencies of the Fe─H bond are influenced by interactions between the amine base of the catalytic cofactor and the conserved cysteine involved in proton transfer to the active site. These findings suggest that H_hyd_ represents the catalytic state just before H_2_ formation.^[^ ^35^ ^]^

**Figure 5:**
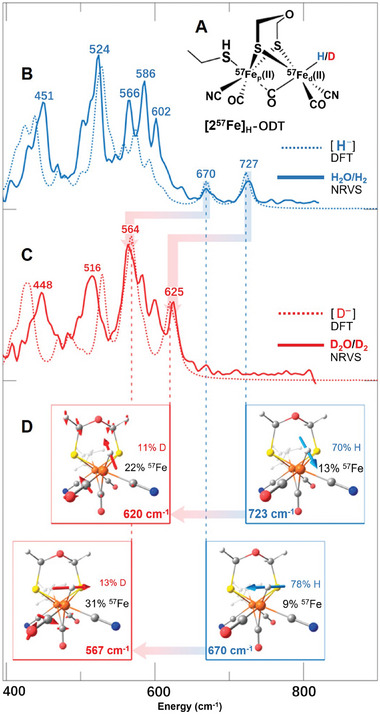
Experimental and DFT calculated NRVS spectra of *Cr*HydA1 maturated with [2^57^Fe]_H_‐ODT. A) Schematic representation of the [2^57^Fe]_H_‐ODT DFT model. B,C) NRVS spectra (solid lines) of *Cr*HydA1([2^57^Fe]_H_‐ODT) in the 400–800 cm^−1^ range recorded on samples in (B) H_2_O/H_2_ (blue) and (C) D_2_O/D_2_ (red), shown overlaid with the corresponding DFT spectra (broken lines) of [2^57^Fe]_H_‐ODT; the NRVS bands are labeled with their positions (cm^−1^). D) Representations of the DFT δ(^57^Fe_d_–H/D) normal modes assigned to the individual NRVS bands; mode frequencies and contributions (%) to the vibrational energy from the ^57^Fe/H/D nuclei are given. The calculated NRVS band positions were scaled with a factor of 0.96. Reproduced with permission.^[^ ^68^ ^]^ Copyright 2017, American Chemical Society.

Direct observation of an iron‐bound terminal hydride in [FeFe]‐hydrogenase was realized by combining FTIR, NRVS, and DFT calculations on an [FeFe]‐hydrogenase variant. Cramer et al. adopted a strategy to accumulate the hydride species in high concentration on an [FeFe]‐hydrogenase variant lacking the amine proton shuttle.^[^ ^68^ ^]^ The NRVS measurements and DFT analysis unambiguously verified the presence of a Fe_d_‐H species in the H_hyd_ state. This species was characterized by a reduced [4Fe‐4S] cluster connected to the oxidized binuclear subsite, Fe(II)Fe(II). DFT calculations explained the nature of the higher energy features observed in the NRVS spectra. Specifically, the NRVS bands at 727 and 670 cm^−1^ corresponding to the H_2_O/H_2_ sample were assigned to bending modes of the ^57^Fe_d_‐H unit. DFT calculations predicted that these modes have ≈80–90% δ(Fe_d_‐H) contribution at 723 and 670 cm^−1^, with hydride motion either in or perpendicular to the Fe_p_‐Fe_d_‐H plane, as depicted in **Figure** **5**.

**Figure 6:**
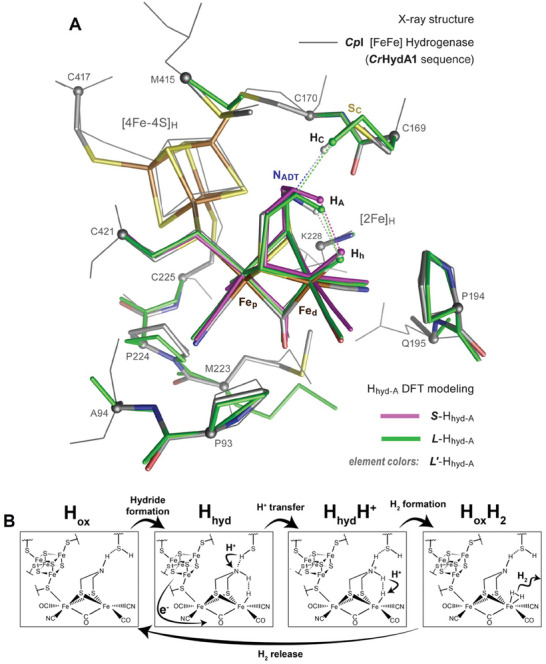
DFT modeling of the H_hyd_ state alternatives. A) The best‐fit H_hyd‐A_ state optimized at three different modeling levels *S* (purple tubes), *L* (green tubes), and *L*′ (tubes in element colors), overlaid with the X‐ray crystal structure^[^ ^70^ ^]^ of the *Cp*I [FeFe]‐hydrogenase (gray wire frame). All protons except the mechanistically central protons (H_h_, H_A_, and H_C_) are omitted for clarity. B) Proposed sequence of events leading to H_2_ formation: protonation of H_hyd_ leading to H_hyd_H^+^, followed by bond formation to give the H_2_ product complex. H_2_ release generates the H_ox_ state of the enzyme, which can be reduced and protonated to form H_hyd_ again. Reproduced with permission.^[^ ^35^ ^]^ Copyright 2017, American Chemical Society.

DFT modeling was also employed to investigate the conformation and protonation state of the bridging ADT ligand and the electronic configuration at the Fe sites in the H_hyd_ state.^[^ ^35^ ^]^ The models incorporated the influence of the protein environment surrounding the H‐cluster, the [4Fe‐4S]H subcluster, and the H‐bonding between ADT protons and the neighboring Cys169/178 residue.^[^ ^69^ ^]^ Two models were used: *L*‐H_hyd_‐A, which includes the integrated protein side chains surrounding the [2Fe] subcluster, and *L'*‐H_hyd_‐A, which additionally includes the [4Fe‐4S]H subcluster (**Figure** **6**A).^[^ ^35^ ^]^

The computational results predicted a H_h_···H_A_ distance of 2.01 Å (**Table** **1**), which closely resembles the Fe─H···H─N distance of 1.88 Å observed in the [(H)Fe_2_(adt‐NH_2_)(CO)_2_(dppv)_2_](BF_4_)_2_ complex determined by X‐ray crystallography.^[^ ^3^ ^]^ Notably, the hydride state in [FeFe]‐hydrogenase is extremely sensitive to subtle effects, such as the hydrogen bonding between Fe─H and ADT and between ADT and Cys169/178. Therefore, the Fe─H···H─N distance is probably not solely determined by the stereoelectronic strength of the interaction. Instead, it is influenced by a complex interplay of the arrangement and orientation of the surrounding atoms/groups. However, this distance is still longer than the ≈1.5 Å H···H distance reported by Bullock and coworkers for a strong Fe─H···H─N dihydrogen interaction.^[^ ^71^ ^]^ The elongated Fe─H···H─N distance in the complex can be attributed to hydrogen bonding with the BF_4_^−^ anion.^[^ ^22^ ^]^ Consequently, the *L*‐H_hyd_‐A model likely represents nuclear motions associated with a step preceding the final step leading to H_2_ formation. The production of H_2_ from the H_hyd_‐A state necessitates the transfer of the amino proton to form a Fe_d_‐H_2_ complex (Figure 6B). This process relies on the assistance of the proton transport chain, either for the initial generation of ‐NH_2_^+^ or for the concerted transfer of H^+^. DFT modeling *L*′ including both [2Fe] and [4Fe‐4S] subclusters indicates that protonation of H_hyd‐A_ yields an [4Fe‐4S]^2+^–Fe_p_(I)Fe_d_(II) species H_hyd_H^+^.

**Table 1:** Important internuclear distances in four alternative H_hyd_ states before H_2_ formation according to the structures optimized by DFT modeling.

DFTModel	Internuclear Distance [Å], *L*‐Modeling				
	Fe_d_− H_h_	H_h_⋅⋅⋅H_A_ / H_E_	N_ADT_− H_A_ / H_E_	N_ADT_⋅⋅⋅H_C_	S_C_⋅⋅⋅H_A_ / H_E_
*L*‐H_hyd‐A_	1.52	2.01 / NA	1.03 / NA	2.26	3.54 / NA
*L*‐H_hyd‐E_	1.51	NA / 3.53	NA / 1.02	3.64	NA / 3.34
*L*‐H^*^_hyd_H^+^	1.53	1.68 / 3.35	1.06 / 1.04	3.78	3.60 / 2.62
*L*‐H_hyd_H^+^	1.58	1.38 / 3.09	1.11 / 1.04	3.89	3.82 / 2.54

**Figure 7:**
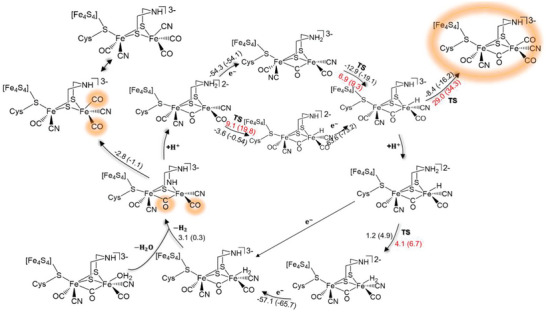
Reaction mechanism calculated with BP86/def2‐TZVP/COSMO and B3LYP/def2‐TZVP/COSMO in parentheses (*ε* = 4). Energies are given in kcal mol^−1^. Reaction energies are shown in black numbers. Reaction barriers are given in red numbers. Note that the surrounding amino acid residues of the model active site have been omitted for the sake of clarity. The charge given is the charge of the full model. Reproduced with permission.^[^ ^31^ ^]^ Copyright 2013, Royal Society of Chemistry.

Synthetic model studies have suggested that a terminal hydride and the ADT cofactor greatly facilitate the reduction of protons to form H_2_ by diiron complexes.^[^ ^65^ ^]^ In the catalytic cycle of [FeFe] hydrogenases, forming intermediates with both terminal and bridging hydride ligands is possible, potentially leading to H_2_ formation.^[^ ^72^ ^]^ The entire proton reduction mechanism in [FeFe] hydrogenases was investigated using a large quantum mechanical active site model embedded in a dielectric continuum, considering the interaction between the H‐cluster and its protein environment.^[^ ^31^ ^]^ Assuming a mechanism involving reactions solely at the terminal position of Fe_d_, hydride formation is found to be exothermic with a small barrier of 6.9 kcal mol^−1^. The subsequent H_2_ formation step also exhibits a low barrier of 4.1 kcal mol^−1^ and is driven by reduction (**Figure** **7**). However, the formation of the thermodynamically more stable µ‐bridging hydride species is kinetically hindered due to a significant overall barrier of 29 kcal mol^−1^. The authors emphasize that the arrangement of amino acid residues in the active site plays a critical role in preventing µ‐hydride formation and enabling efficient proton reduction with low barriers. Moreover, experimental data on a biomimetic complex [Fe_2_(S_2_C_2_H_4_)(µ‐CO)(H)(CO)(PMe_3_)_4_]^+^ demonstrated that a terminal hydride can exhibit higher reactivity than a bridging hydride.^[^ ^65^ ^]^

DFT methods offer a good balance between accuracy and computational cost compared to other methods. While DFT can provide results ranging from poor to fairly good accuracy, depending on the basis set and density functional choice, the widely used B3LYP hybrid functional has demonstrated high accuracy across a broad range of organic compounds. However, improving the quality and accuracy of DFT calculations often comes at the expense of increased computational demands, leading to an ongoing effort in the research community to develop new and more efficient density functionals.

The energetics of proton reduction in [FeFe]‐hydrogenase have also been explored.^[^ ^73^ ^]^ It has been experimentally observed that bridging CO is retained during the catalytic process.^[^ ^35^, ^36^, ^68^, ^74^, ^75^ ^]^ The energy for the bridging hydride structure is significantly lower than that of the experimentally observed structures. Therefore, the proposed model excludes the possibility of a bridging hydride. Considering an H‐cluster charge of −3, the proposed mechanism aligns with available experimental thermodynamics and kinetics. However, the primary challenge lies in the first reduction step, i.e., the formation of H_red_H^+^. Two different experimental suggestions have been made regarding the protonation site in this reduction: one suggests protonation of the nitrogen of the bridging dithiolate, while the other proposes protonation of cysteine on the [Fe4S4] cluster.^[^ ^7^, ^76^ ^]^ The calculations strongly favor the formation of a terminal hydride on the distal iron (Fe_d_) of the [2Fe] dimer in the first reduction.^[^ ^73^ ^]^ In the second reduction, forming H_hyd_, the preferred protonation site is one of the cysteines on the [4Fe‐4S] cluster, which is consistent with the experimental suggestion by Haumann et al.^[^ ^7^, ^76^ ^]^

**Figure 8:**
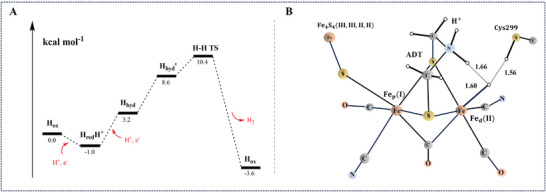
A) Energy diagram of the mechanism for reduction of protons in the case of a blocked bridging hydride, using 15% exact exchange and a charge of the H‐cluster of −3. B) Transition state for the H─H bond formation with a charge of −3 for the H‐cluster. The oxidation state of the iron dimer is Fe(II)Fe(I). The total spin multiplicity is a doublet, and the charge of the H‐cluster is −3. Reproduced with permission.^[^ ^61^ ^]^ Reproduced with modifications. Copyright 2006, American Chemical Society.

The preferred transition state (TS) for H─H bond formation yields another unexpected result. Before reaching the TS, the proton migrates to the nitrogen of the bridging dithiolate, forming H_hyd′_. Interestingly, H_hyd′_ exhibits a unique structural feature with two unusually short hydrogen bonds to the terminal hydride: one from the proton on the nitrogen of the dithiolate and the other from the proton on Cys299. The proton on the nitrogen of the dithiolate appears to play no role in H_2_ formation, and the slightly preferred possibility involves the formation of the H‐H bond between a proton on Cys299 and the terminal hydride (**Figure** **8**). The calculated barrier for this process is relatively low, with only 11.4 kcal mol^−1^.^[^ ^73^ ^]^

**Figure 9:**
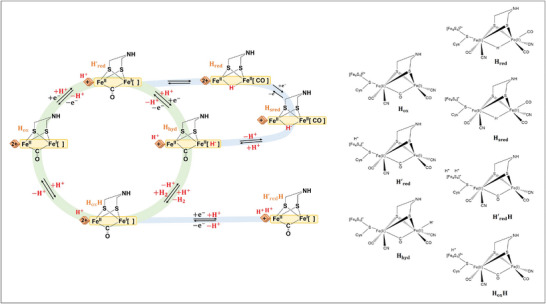
The catalytic cycle of [FeFe] hydrogenases. The H_red_ and H_sred_ states are bridging hydride‐containing species that form a slow catalytic pathway (grey), while H_ox_, H'_red_, H_hyd_ and H_ox_H form the active pathway (blue). Importantly, H'_red_, H_hyd_ and H_ox_H all have protonated forms of [4Fe‐4S]. H'_red_H is formed from H_ox_H by PCET at [4Fe‐4S], yielding a doubly protonated [4Fe‐4S].

However, Haumann et al.^[^ ^7^, ^76^ ^]^ conducted experiments using infrared spectroscopy and isotope editing and found that the nitrogen of the dithiolate ligand was not protonated in any intermediate. Instead, they observed protonation of the [4Fe‐4S] cluster, which was suggested to stabilize a reactive terminal hydride. An alternative model for the catalytic cycle of [FeFe] hydrogenases begins with the active oxidized state, H_ox_ (**Figure** **9**). Proton‐coupled electron transfer (PCET) converts Hox to H'_red_, which is formed by the one‐electron reduction of H_ox_, with the charge residing at the [4Fe‐4S] cluster. In this model, it is proposed that the proton binds to the [4Fe‐4S] subcluster. From H'_red_, the cycle branches into two possible pathways. The first pathway is the active path, where H'_red_ converts directly into H_hyd_. The second pathway involves the µH^−^‐containing H_red_ and H_sred_ states (referred to as H_red_H^+^ and H_sred_H^+^), representing a low‐activity path that the enzyme aims to avoid. On the active path, PCET at the [2Fe] subcluster facilitates the direct conversion of H'_red_ to H_hyd_, bypassing H_red_ and H_sred_. H_hyd_ possesses a terminal hydride on Fe_d_ and is proposed to retain the regulatory proton at the [4Fe‐4S] site. Protonation of H_hyd_ triggers the evolution of H_2_ and results in the formation of H_ox_H. In this oxidized state, the regulatory proton at the [4Fe‐4S] site remains (as shown in Figure 5).

## 4 Concluding Remarks

This review paper provides an overview of the computational modeling techniques employed in the study of [FeFe]‐hydrogenases. There are several relevant quantum chemistry techniques for further study of hydrogenase catalysis. Ab initio wavefunction methods like MP2 and CCSD(T) can provide highly accurate energetics and electronic structures in single‐reference calculations but have a high computational cost that scales poorly with the system size. For a multi‐reference system, calculations with a complete active space multiconfiguration SCF (CASSCF) are highly recommended. However, due to the exponential scaling of an exact correlated calculation with system size, the maximum active space size of a CASSCF is ≈16 electrons in 16 orbitals, although recent developments have made it possible to deal with 20 electrons in 20 orbitals on massively parallel machines. On the other hand, DFT approaches offer a more favorable balance between accuracy and efficiency, though the choice of exchange‐correlation functional is critical. Hybrid quantum mechanics/molecular mechanics (QM/MM) models provide a means to study large enzyme systems by treating the active site quantum mechanically coupled to a cheaper molecular mechanics forcefield for the environment. While requiring careful parameterization, QM/MM can be a powerful tool for investigating reaction mechanisms. Ultimately, the most appropriate computational method will depend on the specific research goals and available resources, with a judicious combination of complementary techniques likely to yield the most comprehensive insights into hydrogenase catalysis.

By combining experimental data with computational insights, researchers deeply understood the mechanisms underlying the catalytic activity of [FeFe]‐hydrogenase and identified challenges and future directions in the field. To advance our understanding, future experiments should establish links between data obtained under different conditions, such as ambient or cryogenic temperatures, and utilize isolated enzymes or whole cells. It is crucial to investigate possible intermediate species between known states.

In addition to steady‐state measurements, studies must be supplemented by transient spectroscopy studies and high‐level quantum mechanical calculations. This combination will help validate the catalytic significance of identified states and provide comprehensive insights into the reaction mechanisms involved. Furthermore, computational studies can help predict the properties and behavior of [FeFe]‐hydrogenases under different conditions. Computer models can simulate the behavior of enzymes and predict their performance by considering factors such as pH, temperature, and substrate concentration. This predictive capability is valuable for optimizing catalyst design and guiding experimental work. The insights gained from these studies can inform the rational design of novel catalysts inspired by [FeFe]‐hydrogenases. The aim is to establish predictive design principles by advancing our understanding of the mechanisms underlying [FeFe]‐hydrogenases and developing computational tools to link their physical properties to catalytic activity. These principles could then guide the development of superior catalysts.

Hydrogen is relatively easy to produce via water electrolysis and is used in fuel cells to build a renewable energy system. However, most hydrogen is generated through steam reforming of natural gas, emitting undesirable CO_2_ byproducts. Water electrolysis is an excellent alternative, but existing electrolyzers require expensive noble metals to achieve high efficiency. Other catalysts, such as Ni‐Fe oxides, necessitate large overpotentials, leading to energy waste as heat. In contrast, the enzyme hydrogenase employs earth‐abundant metals (Ni and Fe) at its active site to efficiently and reversibly reduce protons to hydrogen at low overpotentials. We are expecting more efficient catalysts can be designed based on earth‐abundant materials by studying how the hydrogenase protein environment tunes these metals' properties.

## Conflict of Interest

The authors declare no conflict of interest.
